# Personality Traits and Fatigue in Multiple Sclerosis: A Narrative Review

**DOI:** 10.3390/jcm12134518

**Published:** 2023-07-06

**Authors:** Alessia Ciancio, Maria Claudia Moretti, Antimo Natale, Alessandro Rodolico, Maria Salvina Signorelli, Antonino Petralia, Mario Altamura, Antonello Bellomo, Aurora Zanghì, Emanuele D’Amico, Carlo Avolio, Carmen Concerto

**Affiliations:** 1 Department of Clinical and Experimental Medicine, Psychiatry Unit, University of Catania, 95123 Catania, Italy; alessia.ciancio@gmail.com (A.C.); antimo.natale@yahoo.it (A.N.); alessandro.rodolico@me.com (A.R.); maria.signorelli@unict.it (M.S.S.); petralia@unict.it (A.P.); c.concerto@policlinico.unict.it (C.C.); 2 Department of Clinical and Experimental Medicine, University of Foggia, 71122 Foggia, Italy; maria.moretti@unifg.it (M.C.M.); mario.altamura@unifg.it (M.A.); antonello.bellomo@unifg.it (A.B.); 3 Department of Medical and Surgical Specialities, University of Foggia, 71122 Foggia, Italy; emanuele.damico@unifg.it (E.D.); carlo.avolio@unifg.it (C.A.)

**Keywords:** multiple sclerosis, personality traits, fatigue, neurodegenerative disease, big five factor

## Abstract

(1) Background: Multiple sclerosis (MS) is a chronic neurodegenerative autoimmune disease. Fatigue is a prevalent and debilitating symptom that significantly impacts the quality of life of these patients. A relationship between personality traits and fatigue in MS has been hypothesized but not clearly defined. (2) Methods: A literature search was carried out from databases up to April 2023 for studies correlating personality traits and fatigue in patients suffering from MS. (3) Results: A total of ten articles was included; most of the studies depict a neuroticism–fatigue correlation; however, they were not consistent in terms of the fatigue, personality, and covariate assessments. (4) Conclusions: The clinical and methodological heterogeneity of the included studies prevented us from drawing any firm conclusion on the link between personality traits and fatigue in MS. Several models of personality and different fatigue assessments have been found. Despite this, a common pathway shows that the neuroticism trait or similar personality patterns has a role in fatigue diagnosis. This may be a useful target to improve the quality of life and enhance the modification of the disease treatment results. Further homogeneous and longitudinal studies are needed.

## 1. Introduction

Multiple sclerosis (MS) is an autoimmune-mediated neurodegenerative disease of the central nervous system (CNS) characterized by inflammatory demyelination with axonal degeneration [1]. MS typically affects young adults (mean age of onset, 20–30 years) but the rate of late-onset disease is also growing, and it is characterized by a peculiar course and therapeutic approach [2,3,4,5,6,7].

MS can lead to physical disability, cognitive impairment, and a decreased quality of life and its manifestations include a variety of symptoms. These can include “invisible” symptoms not externally evident to others, such as fatigue, mood disorders, cognitive impairments, pain, bladder/bowel dysfunction, sexual dysfunction, and vision changes [7,8,9,10,11,12]. These symptoms significantly impact the subject’s life and negatively affect daily activities, work, family interactions, and social life [13,14]. The therapeutic armamentarium has grown substantially in the last few years. New high-efficacy therapies also lead to an increased risk of infections or cancers due to the prolonged immunosuppression, especially since they are chronic and lifelong therapies [15,16,17,18].

The therapeutic choice is strictly personalized and is based on demographical, clinical, and radiological characteristics [19,20,21,22,23,24,25]. Biomarkers that can predict disability progression, monitor ongoing disease activity, and assess the treatment response are integral in making important decisions regarding MS treatment [26,27,28,29]. Previous studies [10,30,31,32] have identified fatigue as a key and prevalent symptom in MS, with a greater prevalence of approximately 80% in the primary progressive form compared with the relapsing–remitting form’s prevalence of about 60% [33]. Fatigue is characterized by an overwhelming sense of tiredness, a lack of energy, and a feeling of exhaustion compared to the actual level of activity exerted [34,35]. It is defined as “a subjectively perceived lack of physical and/or mental energy that interferes with habitual and desired activities” [35,36]. It does not appear to be correlated with sex [37] or disease severity; it can be found from the early stages of the disease [38] and it can sometimes be the most important symptom in relapses [39]. Research has also shown that there is a negative correlation between fatigue and the level of disability measured with the Expanded Disability Status Scale (EDSS), resulting in higher levels of fatigue for patients with lower levels of disability [35]. The pathophysiological mechanisms of fatigue in MS are complex and multifaceted [32]. It is currently thought that fatigue arises from multiple different underlying mechanisms, which can make treatment difficult and often results in a trial-and-error approach [32,40]. Many theories have been formulated as hypoactivity of the hypothalamic–pituitary–adrenal axis, autonomic nervous system alterations characterized by sympathetic overactivity and low vagal tone, as well as immune abnormalities (an abnormal imbalance between pro- and anti-inflammatory cytokines) [41,42]. Fatigue has been also related to higher white- and grey-matter atrophy and higher lesion load on magnetic resonance imaging, whilst female sex and higher levels of education seemed to have a protective role towards fatigue [43].

Fatigue strongly affects the quality of life of MS patients, limiting them in daily activities, relationships, and work and thus their lowering quality of life; moreover, it is often found in association with psychiatric symptoms such as depression, anxiety, and insomnia, but also with pain [10,30,31,44]. Various factors such as sleep disturbances, endocrine dysfunction, and mood disorders can contribute to the development or exacerbation of fatigue, necessitating careful investigation for the potential underlying causes [45]. The association with anxiety and depression could be explained by the underlying pathogenic mechanisms such as alteration of HPA [46] and noradrenergic pathways as well as the involvement of pro-inflammatory cytokines [30,47,48,49].

Fatigue is a persistent symptom in MS. In fact, despite the success of disease-modifying therapy in decreasing inflammation, no effects on fatigue have been reported [50]. A recently approved disease-modifying therapy for MS, ponesimod, was compared to teriflunomide in the Oral Ponesimod Versus Teriflunomide In Relapsing Multiple Sclerosis (OPTIMUM) trial [51]. In addition to standard MS treatment measures such as disability and relapse rate, participants in the study were evaluated using a specially developed scale called the fatigue, symptom, and impact questionnaire-RMS (FSIQ-RMS) [51]. Here, the mean difference in FSIQ-RMS, −3.57 (−0.01 vs. 3.56; *p* < 0.001) [51].

About symptomatic treatments targeting fatigue, it has been proposed that fatigue may improve with a different, non-pharmacological or psychological approach rather than specific neurological therapies [52]. Among the pharmacological treatments of MS-related fatigue, methylphenidate, modafinil, and amantadine are commonly prescribed medications for alleviating fatigue in multiple sclerosis (MS) [53]; however, the evidence supporting their efficacy is sparse and conflicting [53,54,55].

Among neurological deficits that may occur in people with MS, neuropsychiatric changes are common and personality disturbances are found in 20–40% of people with MS, including social inappropriateness, disinhibition, apathy, emotional instability, and impulsivity [56]. These personality disturbances affect all aspects of social, personal, and professional activity, treatment compliance, and quality of life (QOL) [57].

Few studies have evaluated personality traits in people with early or early-diagnosed MS. Baseline personality traits are important to recognize early in the diagnosis of MS since they may have a negative impact on the progression of neurological symptoms [58]. Indeed, it is well-known that personality traits may play a pivotal role in the acceptance of the illness and in adaptive coping mechanisms [59,60,61,62] and several studies have considered traits to investigate patients’ responses to disease [63,64,65]. Personality traits reflect people’s characteristic patterns of perceiving, relating to, and thinking about the environment and oneself and they are relatively stable over time [66,67]. 

In clinical research, personality traits are characterized through different psychological models. The theory of Hans Eysenck [68,69] suggests that personality differences are determined genetically, therefore attributing a pivotal role to temperaments and recognizing three dimensions: extroversion/introversion, neuroticism/stability, and Psychoticism/socialization (PEN). Millon’s psychosocial theory [70] focusses on a personality model defined by constitutional elements and past experiences. Such styles might become pathological due to a poor ability to withstand stress, to poor adaptive flexibility, and to a tendency to repeat harmful dynamics without the ability to learn from experience. Psychopathology could derive from the combination of maladaptive coping mechanisms and the associated interpersonal relationships [71]. According to Cloninger’s theory [72], personality is naturally broken down into the psychobiological dimensions of temperament and character; this model proposes that personality is strongly influenced by both genetic (temperament) and environmental (character) variables. The five-factor model of personality [73] identifies the following five personality traits: neuroticism, extraversion, agreeableness, conscientiousness, and openness. In MS patients, a high level of neuroticism and a low level of extraversion and conscientiousness [74] have been observed. Moreover, these traits have been associated with a greater risk of depression and symptoms of anxiety [75], with less physical activity [76], a more cautious approach to life associated with dysfunctional behaviours and lower levels of resilience with dysfunctional coping mechanisms and a higher risk of psychiatric disorders [59]. 

Personality traits and MS have been studied, but no clear relationship between personality and overall function in MS has been described. For these reasons, we aimed to describe the state-of-the-art understanding of this area of MS investigation. In detail, we focussed our attention on personality factors and the perception of fatigue in people with MS.

## 2. Methods

We carried out a search on PubMed and Web of Science until 12 April 2023 by using the following search string: (“multiple sclerosis”[All Fields] AND “fatigue”[All Fields] AND (“personality”[All Fields] OR “personality traits”[All Fields] OR “Five factor model”[All Fields] OR “Temperament and Character Inventory”[All Fields] OR “TCI-R”[All Fields] OR “FFM”[All Fields] OR (“mmpi”[All Fields] OR “mmpi”[All Fields]) OR “PID”[All Fields])). The eligibility criteria were as follows: (1) studies correlating personality traits and fatigue on pwMS; (2) studies written in English. The exclusion criteria were: (1) non-peer reviewed data (e.g., abstracts or trial registry repositories); (2) any other publication different from research studies (e.g., review, case report, commentary, editorial). The relevant data were extracted in a predefined form. The following characteristics were collected: first author and year of publication, country where the study was conducted, study design, MS population characteristics including the EDSS mean measure, personality trait model/tool used, fatigue scale assessed, other clinically relevant data measured, and main results.

## 3. Results

**Figure 1:**
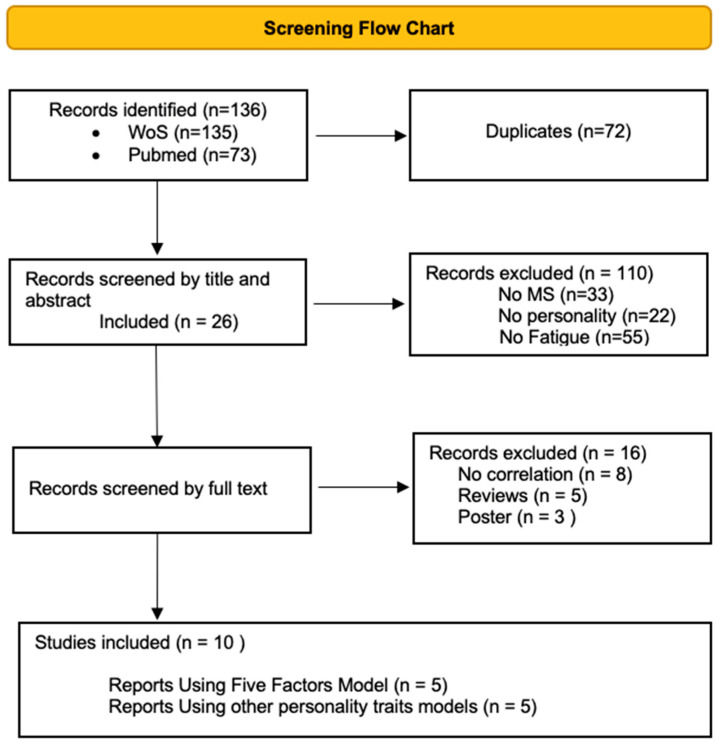
PRISMA flow chart.

The flow chart in Figure 1 shows the screening process. A total of 136 papers was identified. After title and abstract screening, 110 records were excluded, leaving 26 studies. After a full-text screening we included a total of ten studies [77,78,79,80,81,82,83,84,85,86] matching our inclusion criteria. All of them used a cross-sectional study design. Four studies compared findings with a group of healthy controls [77,82,83,84]. The included studies were conducted in various countries (Croatia, Germany, Iran, Italy, the Netherlands, Switzerland, Spain, and the United States). The earliest publication date was 2003 and the most recent was 2021. The most common clinical status of the population studied was relapse–remitting multiple sclerosis (RRMS). Table 1 presents a detailed summary of the core characteristics of the included studies.

Fatigue was measured with different tools valid for MS-related fatigue symptoms. One article [78] assessed fatigue with the Fatigue Impact Scale (FIS) [87], while five studies [77,79,80,82,85] used the modified version of the Fatigue Impact Score (MFIS) [88]. The Fatigue Severity Score (FSS) [89] was administered in three different studies [77,81,82]. Merkelbach et al. [81] added the assessments of the chronic symptomatology with the Chronic Fatigue Scale (CFS) [90] and the subjective consequences of fatigue with the Revised Clinical Interview Schedule (CIS-R). Van der Werf et al. [86] assessed fatigue by using the Fatigue subscale of the Checklist Individual Strength (CIS) [91]. Finally, two studies [83,84] used the Fatigue Scale for Motor and Cognitive functions (FCSM) [92]. Nine studies assessed depression as a confounding factor [77,78,79,80,82,83,84,85,86]. Among them, three evaluated cognition as well [79,84,86].

We found that five studies [78,82,83,84,85] used the five-factor model (FFM) [93,94] to assess personality traits. Among these, Fernández-Muñoz et al. [78] found no significant association between personality traits measured with the NEO Five-Factor Inventory (NEO-FFI) [93] and perceived fatigue measured with Fatigue Impact Scale (FIS) [87]. Penner et al. [82] assessed only the neuroticism and extroversion subscales of the NEO-Five-Factor Inventory, finding that pwMS had a higher score in neuroticism and lower score in extroversion than healthy controls. Despite this result, after controlling for depression, these traits were not related to fatigue, which was assessed with both the Fatigue Severity Scale (FSS) and the Modified Fatigue Impact Scale. In their study, Sindermann et al. [83] assessed personality using the NEO-FFI. High neuroticism predicted cognitive fatigue, while low extroversion was the best predictor for motor fatigue. Spiegelberg et al. [84] confirmed the same finding as Sindermann et al. [83] concerning neuroticism and cognitive fatigue, assessing only the neuroticism subscale of NEO-FFI and comparing it with both FSMC subscales. Strober et al. [85] screened the study population using the neuroticism subscale of the NEO-Five-Factor Inventory-3 (NEO-FFI-3) [95] and the social discomfort subscale of the International Personality Item Pool (IPIP) [96]. PwMS who had higher score on the IPIP social discomfort scale had a T-score greater than 60 on the NEO neuroticism and were identified as having type D personality [97]. This group reported more severe fatigue symptoms compared to pwMS without type D personality.

The included studies evaluated personality traits with different assessment tools. Besharat et al. [77] considered the Skinnerian reinforcement theory (Skinner 1969) to define perfectionism as a personality dimension [98], differentiating two kinds of perfectionism, a positive and a negative (maladaptive) one. The Positive and Negative Perfectionism Scale (PANPS) [99] was administered and was correlated with the total scores of fatigue assessed with MFIS and FSS. Negative perfectionism was directly and significantly associated with MS-related fatigue. Incerti et al. [79] explored personality traits with the Millon Clinical Multiaxial Inventory-III (MCMI-III) [100], following Millon’s model of abnormal personality and coding for DSM IV-TR personality diagnosis. It was found that a higher fatigue score on MFIS correlated with higher scores in several personality traits, such as depression (D), avoidance (Avo), dependence (Dep), and masochism (Mas). Moreover, a higher fatigue score was associated with lower scores in histrionic (His) and narcissistic (Nar) traits. Indeed, the authors noted that fatigue had stronger correlations with clinically significant syndromes such as dysthymia, somatoform disorder, and major depression disorder, as MCMI-III was able to detect them. In their study, Matesic et al. [80] used Cloninger’s psychobiological temperament and character theory for personality through the Temperament and Character Inventory Revised (TCI-R) [101,102]. According to this theory, personality is composed of four temperamental dimensions, heritable and early manifesting, and three-character dimensions, changeable throughout age and life events [101,102]. Their results showed that personality traits directly and indirectly predict MS-related fatigue assessed with MFIS [101,102]. In fact, it was indicated that MS fatigue is dependent upon premorbid harm avoidance. In addition, low self-directedness, as a character dimension, predicted fatigue as well. Merkelbach et al. [81] used the German Freiburg Personality Inventory Revised (FPI-R) [103] tool to assess personality traits, including neuroticism and extroversion. Comparing these two traits with different fatigue assessments tools, such as the Fatigue Severity Scale (FSS) and the Chronic Fatigue Scale (CFS), higher results were found to correlate with higher neuroticism. Moreover, neuroticism has been found to be a strong predictor for severity and chronicity, as their direct correlation with the total scores of both scales showed [81]. Finally, Van der Werf et al. [86] assessed personality traits using the Eysenck Personality Questionnaire (EPQ) [104], focusing on the neuroticism subscale, which result was reported as emotional instability. In their study they correlated through several models the different clinical values with each other, including subjective fatigue assessed with a subscale of the Checklist Individual Strength (CIS-Fatigue) [86]. The findings showed that emotional instability, evidenced by a high neuroticism, led to increased helplessness, which in turn affected fatigue secondarily [86].

## 4. Discussion

This narrative review was conducted to provide an updated summary of the previous evidence on the association between personality traits and fatigue in pwMS. Previous systematic reviews on personality traits and MS have focused on the type of personality traits and how they may impact the primary clinical manifestations and disabilities in people with MS. Limited attention has been given to the association between personality traits and fatigue in MS, and the most recent review on this topic is not up to date [74]. 

From this review, an association between fatigue and personality traits [77,79,80,81,83,84,85,86] emerges, albeit not definitively. The studies under consideration have used different models for the evaluation of personality traits. It is important to proceed with caution when interpreting the data from these studies due to the limited sample size and the varying methodologies used.

Five studies used scales based on the psychological five-factor model [105]. Among them, three [83,84,85] found that neuroticism and/or extroversion were the dimensions that showed a stronger correlation with fatigue.

Elevated neuroticism, defined as behavioural rigidity and a low ability to react to adversity, is the trait most associated with greater levels of fatigue [81,83,84,85,86]. The degree to which basic traits underlie vulnerable narcissism, with a particular emphasis on the importance of neuroticism and agreeableness, has been investigated in a study involving adolescents and young adults [106]; here, analyses demonstrated an association between vulnerable narcissism and neuroticism [106]. This connection between vulnerable narcissism and neuroticism was confirmed by another study indicating that vulnerable narcissism is largely a manifestation of neuroticism [107]. This appears consistent with data in the literature that report that high neuroticism is related to increased somatization and the development of fatigue [76]. Specifically, Strober et al. [85] used a neuroticism subscale from NEO-PPI to determine how many pwMS had a type D personality, defined with high neuroticism and increased psychosocial discomfort [108]. In their results, pwMS scoring higher in neuroticism (hence, included in the type D group) were correlated with more severe symptoms such as increased fatigue and pain, reduced self-effectiveness in disease management, more depressive symptoms, less family support, an increase in perceived stress, and a lower quality of life [85]. 

Two studies have shown that neuroticism is directly associated with fatigue [83,84]. In their findings, neurotic patients tended to report higher cognitive fatigue, defined as a psychological state characterized by feelings of tiredness and impaired cognitive functioning arising from high cognitive demands [83,84]. In general, neurotic people, associated with anxiety, emotional instability, and mood swings, have more problems with concentration and learning new information [109,110]; on the other hand, physical fatigue is related to low extroversion [83]. This may be explained by greater extroversion likely being associated with experiential behaviours that favour the development of physical activity and lead to better physical performance and greater mobility in adults [76].

High neuroticism, strain, and excitability are traits that characterize a high proportion of MS patients (with a higher prevalence in SMRR) and these results are in line with several studies that have reported how disease management from the point of view of thoughts, behaviour, and emotions was unrelated to the type of evolution of MS [111]. In fact, personality traits clearly affect the impact of physical disability, leading to an overestimation of the perception of fatigue, which in turn leads to a worse perception of physical disability [81].

Contrary to what has been reported so far, Penner [82] and Fernandez [78] did not find a direct correlation between high neuroticism and fatigue. 

Fernandez-Munos [78] did not find a correlation between perceived fatigue and personality traits. In fact, they did not find a correlation between fatigue and depression either. They explained this result because of the rarity of depressive symptoms in their sample. This may be said for dysfunctional personality traits as well [78].

Conversely, Penner [82] initially found a correlation between high neuroticism and fatigue; however, it emerged that the only predictors of fatigue were action control and disability by controlling depression as a covariate. These data probably derive from a different methodology implemented in the assessment of depressive symptoms [78]. 

Despite the use of the same personality model, there was no homogeneity in the assessment of depression. These results may be explained by the frequent comorbidity between depression and fatigue in pwMS. 

In the literature, it has been shown that patients with depression present more severe symptoms of fatigue, suggesting that mood disturbance and fatigue are connected to each other in a strong circular relation [112] that cannot be easily discerned by sensitive yet nonspecific self-report tools. 

In fact, Strober [85] does not make any correction of the data based on the depressive symptomatology; Sindermann et al. [83], exclude from their population BDI scores greater than 13; Spiegelberg [84], meanwhile, administered the CES-scaleD, considering it more suitable compared to the BDI scale because it contains fewer physical symptom items and is therefore better able to prevent false positives caused by MS itself. 

It is certain that patients suffering from neurological pathologies have a higher prevalence of developing depressive symptoms [113,114,115] and one of the key physical symptoms of this disorder is precisely fatigue. Penner [82] believes that fatigue can be considered a direct symptom of depression. However, none of this evidence can be replicated or reproduced since different scales of evaluation are used [116].

Fatigue and depression may manifest with the same symptoms, such as a loss of motivation and anhedonia, making these conditions difficult to differentiate [112]. Depressive symptoms should be carefully evaluated using scales that allow the exclusion of the overlap of physical symptoms associated with MS with those arising on a psychological basis [112].

In fact, many of the correlations that emerged from that review were found to be strictly dependent on the different scales used.

In the remaining five articles, personality was assessed through different psychological models with various grades of comparability between each other.

Merkelbach et al. [81] used the FPI-R scale for personality assessment. This scale assessed twelve different personality factors, including neuroticism and extroversion [103]. Both these traits were found to be significantly related to fatigue in MS [81]. They concluded that personality traits clearly impact physical disability, leading to an overestimation of the perception of fatigue, which in turn leads to a worse perception of physical disability [81]. In some parts, their conclusion may be compared to previously commented-on studies even if their assessment is not strictly related to the five-factor model [81].

One study used the Eysenck model [68]. It emerged that elevated neuroticism, understood as emotional instability, is associated with increased feelings of impotence caused by the inability for patients to change their neurological condition. Both MS, as an unpredictable changing stressor, and emotional instability, as personality vulnerability, might lead to higher helplessness, which in turn affects the MS-associated depression and fatigue [68]. 

Hence, higher neuroticism produces more helplessness, which is indeed related to fatigue not only as a direct consequence of depression [86]. In a direct comparison of their models, Costa and McCrae reported that Eysenck’s neuroticism and extroversion are completely consistent with the corresponding factors theorized in the five=-factor model [117].

The literature confirms that high neuroticism and low extroversion are fatigue-related personality traits that could directly determine the fatigue symptom through a patient’s subjective experience of a lack of mental/physical energy, or indirectly by psychological, neuroendocrine, and neurovegetative dysregulation, which would lead to dysfunctional management of the disease (i.e., emotion-centred coping) [42], regardless of the theoretical basis used.

In the study by Matesic et al. [80], the authors investigated the relationship between personality and fatigue in pwMS following the Cloninger theory of temperaments and character. Their results showed that high harm avoidance and low self-directedness directly predicted fatigue, highlighting and underlining how fatigue in MS may be related in part to the premorbid personality. Regarding low self-management, this led to poor management of the disease, leaving patients mentally and physically exhausted [84]. What emerges is that fatigue in MS might stem both from the direct effects of genetically determined temperament, as well as from traits shaped by the environment that develop in later stages of life [84].

Moreover, it emerges that MS patients who have high levels of negative perfectionism and depressive symptoms are correlated with higher fatigue levels [77]. Perfectionism is a personality trait characterized by the search for impeccability and the definition of high standards of performance [98,118]. Two types of perfectionism are recognized, the positive and the negative [119]. In the first case, individuals take pleasure in the search for excellence by recognizing their individual limits, while in the second case the individuals aim to achieve unrealistic objectives and it is therefore considered a mismatching trait [120]. In this article, after correcting for depression, it was evidenced that negative perfectionism correlated with increased fatigue levels, emphasizing how the standards set by individuals with traits of negative perfectionism increase discomfort and somehow lead to an increase in fatigue itself [120]. This appears consistent with data in the literature underlining how the standards set by individuals with traits of negative perfectionism increase discomfort in MS patients, who certainly have limitations associated with pathology [30,121]. 

Moreover, this trait is stressful, characterized by an excessive emotional resonance towards failure, and often aims at implementing behaviours that go beyond the real possibilities. 

Incerti et al. [79] reported that personality traits such as depression, avoidance, addiction, and masochism were related to high fatigue levels. Moreover, histrionicism and narcissism were negatively related to fatigue, unlike in other studies, in which the high neuroticism, loss of empathy, and low pleasantness, elements characterizing high narcissism and histrionicism, correlated directly with fatigue [122,123]. 

However, in our opinion, these data are associated with the use of a scale aimed at diagnosing personality disorders and not at identifying personality traits. In fact, the authors found a correlation of fatigue with diseases such as dysthymia, major depressive disorder, and somatoform disorder. 

Furthermore, this review has highlighted how the studies are uneven in the definition of fatigue. Fatigue is a complex and multidimensional phenomenon that consists of subjective and objective components. In MS, fatigue can be primary or secondary. Primary fatigue is a direct consequence of the disease, while secondary fatigue is a consequence of reduced functional capabilities, chronic pain, and treatment side effects. It may be defined as a decrease in physical and/or mental performance [124]. Nevertheless, the present literature clearly reports that the definition of patients with fatigue often depends on the type of scale used and it might not represent the actual experience of the patient. 

In future studies, homogeneous scales should be used that also allow the differentiation of stretch and state fatigue, such as the FSMC scale and the WEIMuS. 

Despite the great lack of homogeneity, personality has a pivotal role in fatigue symptomatology. However, the studies under consideration are of a cross-sectional design that does not allow the analysis of direct causal links between personality and fatigue. 

Moreover, personality is composed of a premorbid and an experiential part. It should first be assessed whether these dysfunctional personality traits are already present in the premorbid phase, predisposing patients to the development of fatigue, or are the result of maladaptive coping mechanisms and/or the consequence of the inflammatory action associated with the disease. For this purpose, it would be useful to carry out longitudinal studies on naïve patients to analyse over time the direct fatigue–personality causality relationship.

In any case, detecting dysfunctional personality traits may be useful throughout the progression of the neurodegenerative disease to easily achieve and maintain improvements related to disease-modifying therapies.

## 5. Limitations

Medical disabling and progressive conditions could influence personality. No evidence has conclusively shown whether there is a causal link between brain structural damage and personality changes due to inflammation or if these traits are directly related to the MS diagnosis itself. Moreover, anxiety and depression may be considered as confounding factors. Indeed, these symptoms may be higher during active phases or in patients who do not have an adequate MDT response, leading to a worse fatigue experience, regardless of the personality traits presented.

Although the ICD-10 defines a diagnostic personality change (“Enduring personality changes, not attributable to brain damage and disease” F62.0), they were performed to determine whether MS could alter personality due to chronic, persistent pain or because of MS-related psychiatric symptoms (e.g., higher demands of others, higher dependence and expectation).

## 6. Conclusions

This narrative review identified eight studies that found a correlation between personality traits and fatigue. Although some studies have suggested that neuroticism is associated with fatigue in MS patients, these data should be interpreted with extreme caution given the small number of studies under consideration, but especially due to the lack of a common methodology. Future studies should employ unique personality models, utilize fatigue scales with higher specificity and sensitivity indices to increase homogeneity, and carefully study depressive symptoms. This should include removing fatigue-related items from the scales to avoid overlap and eliminate the methodological issues, while also conducting a psychiatric evaluation. Fatigue has been suggested by many authors as a “marker” of disease activity, progression, and prognosis. Thus, it is important to define the role of personality traits, especially because no univocal way to measure them is available and all the data collected are based on self-reported questionnaires.

Given the significant impact of fatigue on the quality of life for MS patients, the limited evidence currently available underscores the importance of further studies. These should aim to identify personality traits that can inform targeted intervention strategies to improve the quality of life of MS patients.

## Figures and Tables

**Table 1:** Studies included and described in the narrative review.

	Personality Trait Assessment	Fatigue Assessment	Other Assessments	Results
Five-Factor Model				
**Penner (2007) [82]** Switzerland Cross-sectional study Participants: 41 Mean Age (SD): 41.80 (10.95) Female/Male: 24/17 MS Diagnosis: McDonald’s criteria Clinical status: 68.3% of patients have RR MS, 7.3% PP MS, 24.4% SP MS EDSS mean (SD): 3.1 (1.71) Years of illness (SD): 8 (7.22) Healthy controls: yes (41 controls)	NEO Five-Factor Inventory (NEO-FFI) N, Neuroticism, A, Agreeableness, C, Consciousness, O, Openness, E, Extroversion (only Neuroticism and Extroversion were assessed)	Fatigue Severity Scale (FSS) Modified fatigue impact scale (MFIS) Scale for Mental fatigue Scale for Physical fatigue	Beck Depression Inventory (BDI) Action control assessment (HAKEMP-90)	MS patients were more neurotic and less extraverted compared to controls; Personality traits not directly related to fatigue after controlling for depression. Comparisons (Cohen’s d) between patients with MS and healthy controls: ○ MFIS: 1.49 ○ FSS: 1.49
**Fernandez-Muños (2015) [78]** Spain Cross-sectional study Participants: 108 Mean Age (SD): 44 (9) Female/Male: 59/49 MS Diagnosis: Modified McDonald’s criteria Clinical status: 74% of patients have RR MS, 8% PP MS, 18% SP MS EDSS mean (SD): 3.6 (1.6) Years of illness (SD): 12.8 (8) Healthy control: No	NEO Five-Factor Inventory (NEO-FFI) N, Neuroticism, A, Agreeableness, C, Consciousness, O, Openness, E, Extroversion	Fatigue Impact Scale (FIS)	Pain Rating scale (NPRS) Functional Assessment of Multiple Sclerosis scale (FAMS) Beck Depression Inventory (BDI-II) Short-Form Health Survey 36 (SF-36)	Personality traits were not associated with self-perceived fatigue.
**Strober (2017) [85]** United States Cross-sectional study Participants: 37 type D pos on 230 pwMS Mean Age (SD): 41.81 (9.82) Type D pos Female/Male: 33/4 Type D pos MS Diagnosis: McDonald’s criteria Clinical status: 97% RR MS EDSS mean (SD): NA Years of illness (SD): 7.18 (7.05) TypeD pos Healthy controls: no	NEO Five-Factor Inventory 3 (NEO-FFI-3) N, Neuroticism, A, Agreeableness, C, Consciousness, O, Openness, E, Extroversion International Personality Item Pool LOC scale (IPIP-LOC) Neuroticism (T > 60) and higher IPIP social discomfort were assessed to determine Type D personality	Modified fatigue impact scale (MFIS) MFIS physical MFiS cognitive MFiS psychosocial	MOS Pain Effects Scale (MOS-PES) Pittsburgh Sleep Quality Index (PSQI) Multiple Sclerosis Self-Management Scale (MSSM-R) Disability Management Self-Efficacy Scale (DMSES) Morisky Adherence Questionnaire (MAQ) COPE inventory General Self-Efficacy Scale (GSE) Chicago Multiscale Depression Inventory (CMDI) State Trait Anxiety Inventory (STAI) Flourishing Scale (FS) Perceived Stress Scale (PSS)	Type D+ individuals were found to report more severe symptoms of fatigue.
**Sindermann (2018) [83]** Germany Cross-sectional study Participants: 52 Mean Age (SD): 45.13 (9.56) Female/Male: 43/9 MS Diagnosis: McDonald’s criteria Clinical status: 54% of patients have RR MS, 17% PP MS, 13% SP MS, 2% CIS, and 13% other. EDSS mean (SD): NA Years of illness (SD): 8.67 (7.36) Healthy controls: yes (screened by BDI-II score < 13)	NEO Five-Factor Inventory (NEO-FFI) N, Neuroticism, A, Agreeableness, C, Consciousness, O, Openness, E, Extroversion	Fatigue Scale for Motor and Cognitive Functions (FSMC) Scale for Cognitive fatigue Scale for Motor fatigue	Allgemeine Depressionsskala (ADS; translated from German as the General Depression Scale)	Low extraversion predicts motor fatigue; High neuroticism predicts for general and cognitive fatigue.
**Spiegelberg (2021) [84]** Germany Cross-sectional study Participants: 30 Mean Age (SD): 46.1 (9.6) Female/Male: 21/9 MS Diagnosis: McDonald’s criteria Clinical status: 80% of patients have RR MS, 13.3% SP MS, 3.3% CIS, and 3.3% other. EDSS median (range): 2.8 (1.5–7–5) Years of illness (SD): 9.1 (8.9) Healthy controls: yes	NEO Five-Factor Inventory (NEO-FFI) N, Neuroticism, A, Agreeableness, C, Consciousness, O, Openness, E, Extroversion (Only Neuroticism was assessed)	Fatigue Scale for Motor and Cognitive Functions (FSMC) Scale for Cognitive fatigue Scale for Physical	Cognitive Failures Questionnaire (CFQ) Center for Epidemiologic Studies-Depression scale (CES-D) Affective Neuroscience Personality Scale (ANPS) (only FEAR subscale was assessed)	High neuroticism had a stronger correlation with cognitive fatigue than with motor fatigue. Comparisons (Cohen’s d) between patients with MS and healthy controls: ○ FSMCc: 1.65 ○ FSMCph: 2.59
**Freiburg Personality**				
**Merkelbach (2003) [81]** Germany Cross-sectional study Participants: 80 Mean Age (SD): 38.50 (9.0) Female/Male: 62/18 MS Diagnosis: Modified McDonald’s criteria Clinical status: 61.25% of patients have RR MS, 13.75% PP MS, 25% SP MS, EDSS mean (SD): 3.2 (1.4) Years of illness (SD): 9.10 (6.20) Healthy control: No	German Freiburg Personality Inventory—revised (FPI-R) LEB, Life Satisfaction SOZ, Social Orientation LEI, Achievement Orientation GEH, Inhibitedness ERR, Excitability AGGR Aggressiveness BEAN, Strain KORP, Somatic Complaints GES, Health Concerns OFF, Frankness E, Extraversion N, Neuroticism	Fatigue Severity Scale (FSS) Chronic Fatigue Scale (CFS)	Revised Clinical Interview Schedule (CIS-R)	Neuroticism was related to fatigue symptoms; Neuroticism was a predictor for more chronic and more severe fatigue.
**Eysenck PEN system for personality**				
**Van Der Werf (2003) [86]** Netherlands Cross-sectional study Participants:89 Mean Age (range): 41.9 (25–69) Female/Male: 63/26 MS Diagnosis: revised Poser criteria Clinical status: 58.4% of patients have RR MS, 41.6% SP MS and PP MS. EDSS mean (SD): 4.4 (1.8) Years of illness (SD): 9.1 (8.9) Healthy controls: No	Eysenck Personality Questionnaire (EPQ) P, Psychoticism E, Extroversion N, Neuroticism Subscale neuroticism was assessed as EI, emotional instability	Subjective Fatigue of the Checklist Individual Strength (CIS-Fatigue).	Illness Cognition Questionnaire (ICQ) Depressed Mood of the Impact of Rheumatic Diseases on General Health and Lifestyle (IRGL),	Emotional instability leads to helplessness, which in turn affected the MS-associated fatigue.
**Skinnerian reinforcement theory**				
**Besharat (2011) [77]** Iran Cross-sectional study Participants: 120 Mean Age (SD): 32.82 (8.22) Female/Male: 79/41 MS Diagnosis: Modified McDonald’s criteria Clinical status: 32.5% of patients have RR MS, 12.1% PP MS, 4.2% SP MS EDSS mean (SD): NA Years of illness (SD): 6.84 (3.99) Healthy control: yes	Positive and negative perfectionism scale (PANPS) PP, positive perfectionism; NP, negative perfectionism	Modified fatigue impact scale (MFIS) Fatigue severity scale (FSS)	Beck depression inventory (BDI)	Negative perfectionism was related to higher fatigue symptoms. Comparisons (Cohen’s d) between patients with MS and healthy controls: ○ MFIS: 1.90 ○ FSS: 1.29
**Millon’s model**				
**Incerti (2015) [79]** Italy D: Cross-sectional study Participants: 77 Mean Age (SD): 43.1 (9.8) Female/Male: 56/21 MS Diagnosis: Modified McDonald’s criteria Clinical status: 82.4% of patients have RR MS, 20.7% SP MS EDSS mean (SD): 3.2 (1.6) Years of illness (SD): 12.9 (7.5) Healthy control: No	Millon Clinical Multiaxial Inventory-III (MCMI-III): 14 personality traits scales 10 Clinical states scales	Modified fatigue impact scale (MFIS)	Full cognitive functions assessment the State Trait Anxiety Inventories Y1 and Y2 (STAI Y1-Y2) Chicago Multiscale Depression Inventory (CMDI)	Depression, avoidance, dependence, and masochism personality traits were directly related to fatigue symptoms; Lower histrionism and narcissism correlated with higher fatigue symptoms.
**Temperament and character theory**				
**Matesic (2020) [80]** Croatia Cross-sectional study Participants: 201 Mean Age (SD): 39.40 (10.81) Female/Male: 153/48 MS Diagnosis: Modified McDonald’s criteria Clinical status: 81.6% of patients have RR MS, 5% PP MS, 10.9% SP MS, 2.5% PR MS EDSS mean (SD): 2.6 (2.12) Years of illness (SD): 7.96 (6.38) Healthy control: No	Temperament and Character Inventory Revised (TCI-R),Four temperament dimensions NS, Novelty Seeking HA, Harm Avoidance, RD, Reward Dependence PS, Persistence. Three character dimensions SelfD, Self-Directedness, CO, Cooperativeness, ST, Self-Transcendence.	Modified fatigue impact scale (MFIS)	Depression scale from the Hospital Anxiety and Depression Scale (HADS)	MS fatigue was to some degree dependent upon premorbid personality; Harm avoidance predicted fatigue; Low self-directiveness predicted higher fatigue.

SD: standard deviation; NA: not available; MS: multiple sclerosis; RR MS: relapsing–remitting multiple sclerosis, PP MS: primary progressive multiple sclerosis, SP MS: secondary progressive multiple sclerosis, CIS: clinically isolated syndrome; EDSS: expanded disability severity score.

## Data Availability

Not applicable.
